# Genito-Urinary Surgery

**Published:** 1905-05-27

**Authors:** 


*(To be continued.)*

---

### GENITO-URINARY SURGERY.

**Tuberculosis of the Kidney.**—Kümmell[1] believes that the best way of curing tuberculosis of the bladder is to remove the kidney responsible for the infection. The bladder should not be operated upon, but the kidney should be removed as soon as possible. The kidney becomes infected through the blood or lymph, and the bladder is infected in turn from the kidney. The prognosis is favourable if the kidney is removed. The writer obliterates the ureter in its entire length by inserting a galvano-cautery which has the shape of a sound, and can be heated for 10 cm. from its tip. It is passed into the ureter from the nephrectomy wound in the cold state, and the current then turned on. Thus complete obliteration of the lumen of the ureter is secured. The ureters are involved very early in the disease, and this may sometimes be recognised by palpation through the vagina or rectum, the ureters being then felt as thickened cords. H. N. Vineberg (New York)[2] points out that primary renal tuberculosis is most frequently met with in women, and that the cases often come first to the gynæcologist. Statistics based on the findings at autopsies show, however, that males are more frequently affected with renal tuberculosis than females. These different findings are explained by the fact that in men the disease may be primarily renal, but is generally of the ascending variety, starting from the genital organs, and hence in them it is not so frequently amenable to surgical intervention. Israel only found one case in literature in which in a female genital tuberculosis was associated with renal tuberculosis. Another example was recorded by Hunner from the service of H. A. Kelly. In both these cases tuberculous adnexa had to be operated upon some years after a tubercular kidney had been treated. Renal tuberculosis is most frequently met with in the third and fourth decades of life. The onset is usually very insidious, and the disease may exist for a long time without causing appreciable symptoms. The first symptoms are generally referable to the bladder, and the patients are treated for cystitis. The urine is usually acid, containing a variable amount of pus and some red corpuscles, but rarely containing casts. The cystoscope may help much in diagnosis, showing, for example, ulceration about one ureteral orifice or scattered tubercles. The author records a case in which an erosion surrounding one ureteral orifice proved not to be of tubercular origin but to be probably due to pressure of the fœtal head during delivery. To discover if the other kidney is healthy the author catheterises the sound ureter by the Kelly method, with the patient in the knee-elbow position, and he considers the risk of infecting the ureter, if suitable precautions are taken, to be very small. The simple and fairly efficient test of Völcker and Joseph is alluded to. These authors use indigo-carmine, 0·4 g.m. freshly dissolved in 10 c.c. of sterile normal salt-solution. Of this solution 4 c.c. are injected deeply into the gluteal muscles. The dye is sterilised by exposure to steam. This dye has the advantage that when it is excreted through the ureters it does not mix readily with the urine in the bladder and can be well seen with the cystoscope. It is suggested that the functional capacity of the kidneys may be estimated by noting the intervals that elapse between the rhythmical ejection of the stained urine from the ureteral orifices. Fortunately in women renal tuberculosis is in the vast majority of cases unilateral. Israel now removes the ureter, even though it appears normal, as well as the affected kidney, using a second short incision parallel with Poupart's ligament for the purpose. The removal of the twelfth rib may greatly help in the removal of a high-lying kidney. Primary nephrectomy yields a smaller mortality than nephrectomy preceded by nephrotomy. Israel's results show a mortality of 10·7 per cent. for the operation and these are probably the best results yet attainable. The ultimate prognosis is good. F. Tilden Brown[3] points out that frequency of micturition is a symptom probably in 90 per cent. of cases of renal tuberculosis. This may be associated with a hyperæmic, œdematous, or ulcerated condition of the ureteral papilla, but this is not always present, and though such a condition may aggravate the frequency of micturition it would seem as if the descent of tuberculous elements in the urine can alone produce the vesical irritability without reflex innervation from the kidney.

**Sarcoma of the Kidney.**—Charles N. Dowd (New York)[4] recently removed a tumour of this nature from a girl six years old. The mass extended from the diaphragm into the left part of the pelvis, bulging the lumbar region and extending across the median line two or three inches. The incision extended from the twelfth rib at the margin of the quadratus lumborum across the median line below the umbilicus. The peritoneum was opened for four inches. The tumour was easily removed and convalescence prompt. Dowd quotes statistics collected by Waller, based on the records of 74 operative cases, which showed an operative mortality of 36·48 per cent., and a mortality within three years of between 73 and 93 per cent. The percentage of patients living beyond the three-year limit was 5·4 per cent. In discussing Dowd's case Lilienthal said the Trendelenburg posture facilitated the operation, and that he had removed two similar growths, one almost of the same size as the above, without opening the peritoneum. Kammerer said he would attack these tumours retroperitoneally but would open the peritoneum to assure himself as to the position of the colon, which could not always be recognised by touch.

**Diseases of the Ureters.**—Dr. Brewer[5] reports the successful removal of a stone in the pelvic ureter (male). The patient had had almost constant pain in the right inguinal region for five months and a few days before operation had severe renal colic for 48 hours. A skiagram showed the presence of a stone. The kidney and ureter were slightly tender. The iliac extraperitoneal route was chosen for the operation. The stone was found one inch below the pelvic brim and removed through an incision in the ureter. Dr. Brewer alludes to a similar case in which operation was refused in spite of repeated attacks of colic, and one night, during an attack of colic, the patient drank quickly two quarts of Poland water and an hour later the stone was expelled. H. A. Fowler (Washington)[6] reports two similar cases. In the first case, that of a man of 36, the complaint was of attacks of hæmaturia and constant desire to urinate. The cystoscope showed the ureteral orifice on the affected side to be small, wizened, non-patulous, and closed by a plug of mucus. The other ureter was acting vigorously. Also the trigone on the affected side appeared to be atrophied. With Casper's catheterising cystoscope an impassable obstruction was found on this side 4 cm. from the orifice. Uretero-lithotomy was successfully performed. In the second case there had been attacks of colic at intervals for 22 years, and for six years there had been increasing trouble from frequency of micturition. An impacted stone was removed successfully as in the last case. Vesical irritability was a marked feature in each case, and in the second one pain at the end of the penis was a symptom. Thus the symptoms when a stone is in the pelvic ureter are like those of a stone in the bladder. The iliac extra-peritoneal route seems to be the best in dealing with these cases by operation. If the stone is in the intra-vesical or intramural portions of the ureter it will be best removed by supra-pubic cystotomy. J. A. Sampson (Baltimore)[7] reports three cases operated upon for diseased conditions of the lower ends of the ureters under local anæthesia (cocaine). He alludes to the great recuperative power of the kidneys, and to the fact that cases have occurred in which both ureters have been accidentally ligated for from 24 to 72 hours, and yet after the ligatures have been removed, or the ureters have been reimplanted into the bladder, the kidney have resumed their function, and the patients have recovered. In the first of the author's cases there was double pyonephrosis associated with inefficiency of the uretral openings into the bladder. These orifices permitted the regurgitation of fluid from the bladder into the ureters and kidneys, as proved by distending the bladder with fluid and in that way producing pain in both kidneys. The ureters were each in turn resected and implanted into the bladder in two operations by the inguinal extra-peritoneal route under cocaine. The duration of each operation was over five hours. The ureter was rendered efficient, that is it remained permeable to urine in the normal direction only, on one side, but on the other side regurgitation still occurred. In the second case the lower end of the ureter on one side was treated as in the last case for a uretero-vesical fistula. The fistula was cured but the implanted ureter apparently became occluded. In the third case a stone was removed from the lower end of the ureter. The effects on the patients of these prolonged operations under cocaine anæsthesia (Schleich's solution), was that of fatigue. The author discusses at length the advantages and disadvantages of operating under local anæsthesia. During parts of the operations some degree of pain was inflicted. General anæsthetics may be contra-indicated in cases of renal insufficiency.

[1] Nova Medica, November, 1904, p. 15. [2] Med. Rec., Nov. 12, 1904, p. 761. [3] Annals of Surg., January, 1905, p. 131. [4] Ibid., October, 1904, p. 595. [5] Ibid., p. 588. [6] Ibid., December, 1904, p. 943. [7] Ibid., February, 1905, p. 216.

